# Feasibility of a Guided Web-Based Procrastination Intervention for College Students: Open Trial

**DOI:** 10.2196/72896

**Published:** 2025-10-16

**Authors:** Sevin Ozmen, Arpana Amarnath, Leonore de Wit, Chris van Klaveren, Pim Cuijpers, Annemieke van Straten, Sascha Struijs, Caring Universities Consortium

**Affiliations:** 1 Department of Clinical, Neuro and Developmental Psychology, Amsterdam Public Health Research Institute, Vrije Universiteit Amsterdam, De Boelelaan 1105, Amsterdam, 1081 HV, The Netherlands, 31 20 598 9898

**Keywords:** adherence, eHealth, guidance, online interventions, procrastination, satisfaction

## Abstract

**Background:**

College students commonly struggle with procrastination, which is linked to mental health complaints and poor academic performance. Interventions based on cognitive behavioral therapy can be effective in reducing procrastination. Traditional face-to-face therapy and online interventions have shown promising outcomes, with the latter overcoming help-seeking barriers such as lengthy referral processes and waiting lists.

**Objective:**

This study aims to examine the feasibility and acceptability of a new eHealth intervention targeting procrastination for college students (“GetStarted”) with guidance by student e-coaches. This cognitive behavioral therapy–based intervention was designed specifically for and together with the target demographic of students studying in the Netherlands. Guidance was offered by trained clinical psychology students in the form of written motivational, supportive messages.

**Methods:**

We conducted a single-arm study. The primary outcomes were satisfaction (8-item Client Satisfaction Questionnaire [CSQ-8]), usability (10-item System Usability Scale [SUS-10]), and adherence (completion rate). The secondary outcomes were changes to procrastination (Irrational Procrastination Scale [IPS]), depression (9-item Patient Health Questionnaire [PHQ-9]), stress (10-item Perceived Stress Scale [PSS-10]), quality of life (Mental Health Quality of Life Questionnaire [MHQoL]), and e-coaching satisfaction (Working Alliance Inventory for Guided Internet Interventions [WAI-I]).

**Results:**

Of 734 participants who started the intervention, 335 (45.6%) completed the posttest. Students reported being satisfied with the intervention (CSQ-8: mean 23.48, SD 3.23) and found it very usable (SUS-10: mean 34.39, SD 4.52). Regarding adherence, participants completed 68.95% of the intervention on average, while 36.65% (n=269) of participants completed the full intervention. Participants showed a significant decrease in procrastination (IPS: mean decrease 35.39-32.56, Cohen *d*=0.63), depression (PHQ-9: mean decrease 9.27-7.73, Cohen *d*=0.35), and stress (PSS-10: mean decrease 20.79-19.02, Cohen *d*=0.31) as well as an increase in quality of life (MHQoL: mean increase 12.81-13.65, Cohen *d*=0.37) from baseline to posttest to follow-up. Participants reported a moderate-to-strong alliance with their e-coach (WAI-I: mean 45.26, SD 7.72).

**Conclusions:**

The internet-based, student-guided intervention “GetStarted” targeting procrastination appears to be acceptable and feasible for college students in the Netherlands. However, high attrition rates and the lack of a control group mean that results must be interpreted with caution. To further examine intervention effectiveness, a randomized controlled trial needs to be conducted.

## Introduction

Procrastination, which refers to the voluntary delay of a necessary course of action despite expecting negative consequences [1], is pervasive among college or university students (henceforth referred to as “college students”). Academic environments, in particular, present unique challenges that could exacerbate procrastination, such as heavy workload, high autonomy, and competing priorities [2-4]. Consequently, studies suggest that 29%-67% of students [5-8] frequently procrastinate, making it a significant concern in higher education settings. In an academic setting, procrastination is associated with poor academic performance with low grades, increased dropout, and academic dishonesty among college students [9-12]. In addition, procrastination has also been linked to several mental health concerns, such as depression, anxiety, stress, low self-esteem, and maladaptive coping [13-17]. Given these associations, it is not surprising that there would be long-term adverse effects of procrastination on overall health, interpersonal relationships, financial stability, and career opportunities [16,18,19].

It is therefore important to find and implement effective treatments that could help students understand and decrease their procrastination behaviors. Meta-analytical evidence shows that effective treatments for procrastination exist [20] and that cognitive behavioral therapy (CBT) was most effective in reducing procrastination in comparison with self-regulation training, strengths training, and acceptance-based behavior therapy [21].

However, accessing mental health care is a challenge for many young people. Unclear access points, lengthy referral processes, waiting lists, and concerns about privacy pose significant barriers [22]. In addition to this, some students who experience problems are reluctant to seek help for various reasons such as perceiving the problem as being temporary or minor, preferring to handle the problem alone or by talking to close ones, a perceived lack of time, fear of stigmatization, and limited awareness of available resources [23-26]. This highlights the need for more accessible, community-based support that is less stigmatized than traditional clinical interventions.

In recent years, eHealth interventions have gained popularity and proved well-suited to college students’ dynamic lifestyles and diverse challenges [27-29]. From accessibility, convenience, cost-effectiveness, and anonymity to the possibilities of personalization, promotion of health literacy, feedback mechanisms, and cultural and linguistic inclusivity, eHealth interventions address many barriers to treatments commonly faced by students [27]. In line with this, some studies have shown that eHealth interventions effectively reduce procrastination among college students [30-32]. Compared to face-to-face, chat-based, and in-person group therapy, eHealth treatment has yielded comparable results in improving procrastination behaviors [33,34].

However, a common problem faced by most of these studies was that of nonadherence. Several eHealth interventions targeting different mental health concerns have had to face this issue of nonadherence with higher dropout rates [35-38] and participants not making optimal use of interventions [39]. This undermines the efficacy of the interventions and reduces their potential to deliver meaningful health outcomes [40]. To add to this, the very nature of procrastination could exacerbate nonadherence as these individuals could continuously put off participating in or completing the intervention [32]. There could be several reasons for nonadherence, such as lack of motivation, engagement [41], or understanding of the platform’s utility [37]. A possible solution could be adding guidance to the intervention [42-44]. Providing structured guidance could enhance user engagement, accountability, and the perceived value of the intervention [45,46]. However, this solution is not always feasible due to the scarcity of mental health professionals [47].

Therefore, it is imperative to form alternate and effective forms of guidance. In recent years, past studies have indicated that eHealth interventions that utilized guidance provided by nonclinicians (eg, peers, research assistants, or other lay persons) were just as effective as those guided by clinicians [48]. This form of guidance can have added benefits by reducing the cost of implementation and improving the scalability, accessibility, and flexibility of the intervention [49]. In addition, laypersons could foster a connection and a positive working alliance by reducing stigma, as the interaction could feel less clinical compared to a professional setting [48]. An available resource that could provide this type of guidance is guidance provided by clinical psychology students. As clinical psychology students receive psychological training during their studies, they will likely provide optimal qualitative guidance. However, to the best of our knowledge, this type of guidance in eHealth interventions for procrastination has not yet been evaluated.

We developed a new online intervention targeting procrastination (“GetStarted”) for and in collaboration with students studying in the Netherlands, addressing the lack of existing interventions specifically designed for this group. This allowed us to ensure that the contents of the intervention were relevant and appealing to this target demographic. The intervention was based on CBT, given meta-analytical evidence that this is the most effective type of intervention [21]. Specifically, the intervention utilized cognitive restructuring, which is a key mechanism of change in CBT [50].

Due to its novel nature, the intervention has not yet been examined in the scientific literature. Therefore, this study evaluates the feasibility and accessibility of a newly developed online intervention guided by psychology student e-coaches to reduce procrastination among college students. The following research questions (RQs) were addressed:

- Main RQs:
-  RQ1: How satisfied are participants with the new online intervention “GetStarted” and its platform?
-  RQ2: What are the adherence rates of the new intervention?
- Secondary RQs:
-  RQ3: What are the differences between baseline to posttest to follow-up for procrastination, depression, perceived stress levels, and quality of life?
-  RQ4: How satisfied are participants with the e-coaching provided by clinical psychology students?

## Methods

### Study Design

A single-arm open trial design was used to assess the feasibility and acceptability of a new eHealth intervention aimed at reducing procrastination among college students, which are the primary aims at this stage. This time- and cost-efficient approach allowed for initial insights into engagement and implementation before proceeding to a randomized controlled trial to better examine effectiveness. In this single-arm within-group design, assessments were administered at baseline (t0), post intervention (t2), and follow-up (t2), occurring at 4 weeks and 6 months post baseline, respectively.

### Ethical Considerations

The ethical approval was granted by the VU Scientific and Ethical Review Board (reference 2020.088). Participants gave their informed consent, and their privacy and confidentiality were respected through the omission of any identifying details. Participants were not offered compensation for their participation. This study is conducted within the Caring Universities Consortium, which comprises 7 Dutch universities (VU Amsterdam, Leiden University, Maastricht University, Utrecht University, Erasmus University, University of Amsterdam, and Inholland University of Applied Sciences). This consortium is affiliated with the World Health Organization’s World Mental Health International College Student Initiative (WMH-ICS) [51].

This study adheres to the STROBE (Strengthening the Reporting of Observational Studies in Epidemiology) guidelines. A completed checklist is provided in Checklist 1.

### Sample Size

There is no standardized method for determining the sample size of an open feasibility study, and previous research has recommended 12 [52] to 35 or more participants [53]. Drawing on these guidelines and comparable studies [54,55], we estimated that 50 participants would be sufficient to address our primary objectives. However, the present intervention was available to the general student population for several years, and we expected that more than 50 participants would sign up. For quality assurance, a power analysis was conducted, and aiming for a conservative 2-tailed calculation with a small effect size (0.2), our statistical analyses (estimating differences between 2 dependent means) would require a total sample size of 199 participants [56]. In this study, we included all participants who had used the intervention and completed the posttest at the time of data analysis to be able to draw firmer conclusions.

### Participants and Enrollment

Participants were students enrolled in any one of the 7 Dutch universities within the Caring Universities consortium. Recruitment took place in three ways from January 2021 to December 2023. First, through an annual online mental health survey of the WMH-ICS, participants who scored above 28 points on the Irrational Procrastination Scale (IPS) in the survey were invited to participate in the intervention. Second, through social media marketing as well as on-campus marketing via the distribution of posters, flyers, and other promotional materials. Third, college staff members such as student psychologists, study advisors, and lecturers could recommend the intervention to students who might benefit from it.

### Eligibility Criteria

Participants were eligible if they were (1) aged 16 years or older, (2) enrolled as a student in one of the participating universities, (3) reported no active suicidal ideation, and (4) provided informed consent.

We identified participants with suicidal ideation with a series of questions. Participants who indicated that they had recently had thoughts of killing themselves and were somewhat likely or very likely to act on these thoughts were excluded from the intervention. These participants were provided with an extensive list of possible resources to turn to. This message was also sent to their email address.

### Intervention

The intervention “GetStarted” was created in collaboration with a User Experience designer and followed the principles of an optimized user interface [57]. Based on the principles of CBT, the intervention consisted of 5 mandatory modules and 4 optional modules. The optional modules became available to participants upon completion of the second mandatory module. Participants could choose whether they wanted to do optional modules, which ones, and in which order. An overview of all the modules can be found in Table 1.

**Table 1.** Intervention content.

Module type	Module name	Contents
Mandatory	You, the monkey and the monster	Introduction to the program and its contents
Mandatory	Protection level: 5000	Understanding the psychological processes behind procrastination: procrastination as an avoidance mechanism
Mandatory	A chat with Procrastination Brain	Identifying the negative thoughts and feelings that accompany certain tasks, causing procrastination
Mandatory	I’ve got to break free	Cognitive restructuring to challenge and change negative thinking patterns
Mandatory	Self high-five	Reflecting on and celebrating one’s progress, summary of all main modules
Optional	Bigger isn’t always better	Breaking a big task into smaller parts and prioritizing or ordering these tasks
Optional	Panic Monster to the rescue	Estimating how much time a task will take, creating a timeline, methods to uphold the timeline
Optional	Focus booster pack	Ways to increase productivity (eg, preparing properly, eliminating distractions, working in time blocks)
Optional	The master of motivation	Motivational techniques (eg, goal setting, visualization, positive thinking)

### Guidance

The intervention was guided by e-coaches. These e-coaches were trained (research) master’s students in clinical psychology and third-year clinical psychology bachelor students. To be eligible for the position of e-coach, students had to have successfully completed courses on diagnostics and psychological interviewing. Selected candidates completed 6 hours of training before commencing their coaching activities.

When participants enrolled for the intervention, they were given the option to choose an e-coach based on a photo and a short biography of 3 random e-coaches. From then on, the e-coaches supported the participants and provided guidance through asynchronous, text-based feedback delivered via the platform. Feedback was written based on the participants’ answers to the questions posed to them by the intervention. In the feedback, coaches showed empathy and understanding, encouraged the participant to apply the newly learned techniques, and asked reflective questions.

These coaches also attended weekly 1-hour intervision sessions under the supervision of the research team to enhance their coaching skills and maintain quality standards. During each intervision session, several e-coaches would share messages they had sent to their participants and would receive feedback from their peers and the supervisor. The supervisor also checked messages sent by e-coaches on the platform on a sample basis as a form of quality control.

### Assessment Measures

#### Primary Outcomes

##### Satisfaction With the Intervention

The 8-item Client Satisfaction Questionnaire (CSQ-8) was used to evaluate participants’ satisfaction with the intervention [58]. It consists of 8 questions on a 4-point Likert scale (sum score 8‐32), and a higher score indicates greater satisfaction. The CSQ-8 has shown high reliability and validity for online interventions [59].

##### Usability

The usability of the intervention was assessed using the 10-item System Usability Scale (SUS-10) [60]. It consists of 10 questions on a 5-point Likert scale (sum score 0‐40). Total scores are then multiplied by 2.5 to achieve a total score of 0-100, where a higher score indicates greater usability. The SUS-10 has shown good psychometric properties (reliability and validity) [61].

##### Adherence

Adherence is “the degree to which the user followed the program as it was designed” [62]. In this study, modules 1‐4 were considered core modules as they contained the CBT elements. Therefore, participants who completed these 4 core modules were considered completers of the intervention. Additionally, average adherence across the total sample was calculated by dividing the number of core modules completed by the total number of core modules in the program and multiplying this by 100. The resulting percentage indicates the adherence rate.

Participants who did not complete a single module were excluded from the analyses as they did not start the treatment.

### Secondary Outcomes

#### Procrastination Tendencies

Procrastination behavior was measured using the IPS [63,64]. It consists of 9 questions on a 5-point Likert scale (sum score 9‐45), and a higher score indicates more procrastination behavior. The IPS showed good internal consistency of Cronbach *α*=0.91 [63]; a high level of reliability (point measure correlation of 0.58‐0.74); and good content, structural, and substantive validity [65].

#### Depressive Symptoms

The 9-item Patient Health Questionnaire (PHQ-9) was used to measure depression [66]. It comprises 9 questions on a 4-point Likert scale (sum score 0‐27), and a higher score indicates a higher level of depression. The PHQ-9 showed high sensitivity (0.71‐0.84), specificity (0.90‐0.97), internal consistency (Cronbach *α*=0.86‐0.89), test-retest reliability (*r*=0.84), and validity [66,67].

#### Perceived Stress

The 10-item Perceived Stress Scale (PSS-10) was used to measure stress [68,69]. It comprises 10 questions on a 5-point Likert scale (sum score 0‐40), where a higher score indicates higher perceived stress. The PSS-10 showed high validity, internal consistency (Cronbach *α*=0.74‐0.91), and test-retest reliability (*r*=0.74‐0.88) [69,70].

#### Quality of Life

The Mental Health Quality of Life Questionnaire (MHQoL) was used to assess quality of life [71]. It comprises 7 questions about different dimensions of life (eg, mood, relationships) on a 4-point Likert scale (sum score 0‐21), where a higher score indicates better quality of life. The MHQoL showed good validity, test-retest reliability (*r*=0.85), and internal consistency (Cronbach *α*=0.85) [71].

#### E-Coach Evaluation

Participants’ satisfaction with the e-coach was assessed using the Working Alliance Inventory for Guided Internet Interventions (WAI-I) [72]. It comprises 12 questions on a 5-point Likert scale (sum score 12‐60), where higher scores indicate higher satisfaction. A score of 36 or higher indicates sufficient satisfaction, where a score of 48-60 means high to very high satisfaction with the e-coach. The psychometric characteristics of the WAI-I yielded adequate results [72].

#### Sociodemographic Information

The following sociodemographic information was collected: age, gender, marital status, nationality, attending university, faculty, education level (bachelor’s, master’s, or PhD), and whether psychotherapy, medication, or both were received. This information was collected, first, to gain insight into the characteristics of individuals who are interested in the intervention when offered access to it in a real-life setting. Second, we wanted to ensure that there were no major differences between participants who did and did not complete the study to account for high attrition rates.

### Assessments

The overview of the assessments can be seen in Table 2.

**Table 2.** Measures and assessment points.

	Assessment points		
	T0^a^	T1^b^	T2^c^
Sociodemographics	✓		
8-item Client Satisfaction Questionnaire		✓	
10-item System Usability Scale		✓	
Working Alliance Inventory for Guided Internet Interventions		✓	
Irrational Procrastination Scale	✓	✓	✓
10-item Perceived Stress Scale	✓	✓	✓
9-item Patient Health Questionnaire	✓	✓	✓
Mental Health Quality of Life Questionnaire	✓	✓	✓

a T0: baseline.

b T1: posttest (4 wk after T0).

c T2: Follow-up (6 mo after T0).

### Data Analysis

IBM SPSS version 27 was used for the data analyses.

First, we examined the baseline characteristics of the whole sample, study completers versus noncompleters (ie, participants who did vs did not complete the posttest assessment) and intervention completers versus noncompleters (ie, participants who did vs did not complete at least 4 main modules). Potential differences in baseline characteristics between these groups were examined using *χ*^2^ tests and independent sample *t* tests.

For our primary outcomes of client satisfaction (CSQ-8) and usability (SUS-10), we conducted a complete case analysis and analyzed only study completers. We calculated descriptive statistics for the whole sample and examined intervention completers versus noncompleters as an additional sensitivity analysis. For the primary outcome of adherence, we calculated how much of the program was completed on average, as well as the percentage of participants who completed the intervention versus did not complete the intervention.

For our secondary outcomes, which were of an exploratory nature, we conducted a complete case analysis and examined whether there was a statistical difference from baseline to posttest in reported procrastination behavior (IPS), depression (PHQ-9), stress (PSS-10), and quality of life (MHQoL). We conducted 2-tailed paired *t* tests using a significance level of *α*=.05 to assess these changes. To interpret the effect size, we calculated the Cohen *d*, interpreting the benchmarks of 0.2, 0.5, and 0.8 as small, moderate, and large, respectively [73].

## Results

### Participants

A total of 1746 students were assessed for eligibility for the intervention between January 2021 and May 2023. Of these, 657 participants did not receive access to the intervention because they dropped out during the baseline assessment (n=204), dropped out during account creation (n=359), or were excluded due to active suicidal ideation (n=94).

A total of 1089 participants were granted access to the intervention. Of these, 355 (32.6%) did not start the intervention. The reasons for not accessing the intervention were not activating their account from a link they received via email (n=121) or completing zero modules within the intervention and therefore not starting the treatment (n=234). There were no baseline differences between those who started the intervention and those who did not.

**Figure 1.**
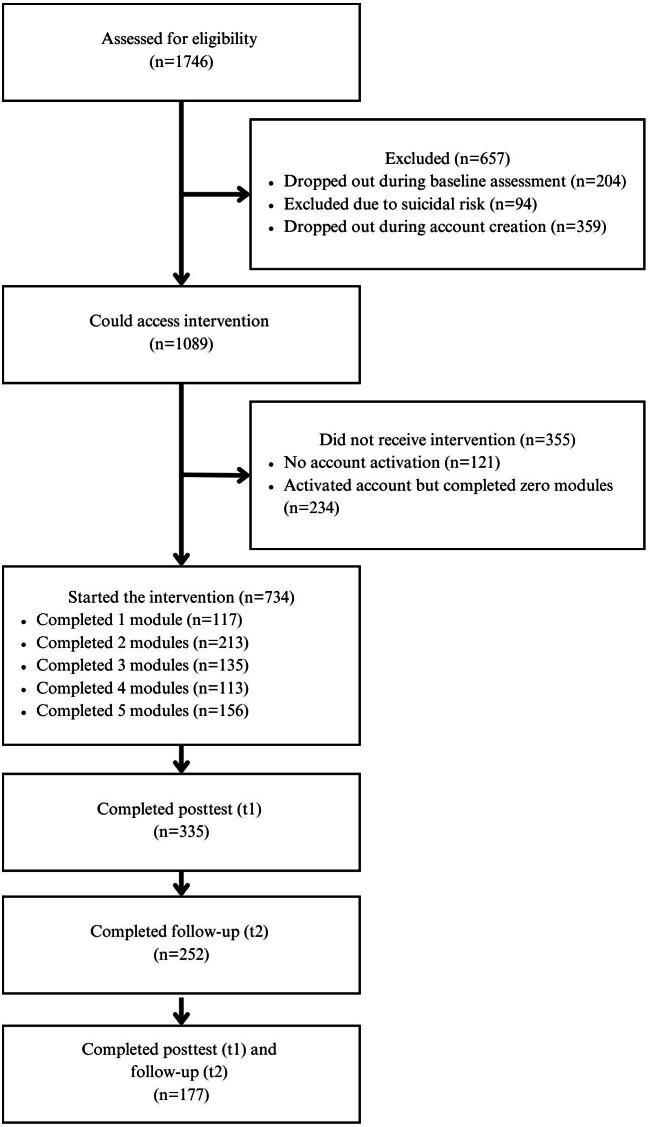
Flow diagram of participants.

A total of 734 participants started the intervention. Our analyses on adherence were conducted based on this sample. Of these 734 participants, 335 completed the posttest (ie, study completers) and were included in the satisfaction and clinical outcome analyses. The details on the participant flow can be found in Figure 1.

The average age of the total sample (n=734) was 23.6 (SD 4.15) years, and most participants were female (n=543, 74%). Most were from the Netherlands (n=446, 60.8%) or another country in Europe (28.6%). Almost half of the participants were currently doing their master’s (n=340, 46.3%). A vast majority of participants (n=615, 83.8%) were currently receiving no medication or psychotherapy. We compared baseline characteristics of intervention completers and noncompleters as well as study completers and noncompleters. Age, gender, nationality, university, education level, marital status, current professional help, and baseline clinical characteristics (procrastination, depression, stress, and quality of life) were compared. We found no significant differences between intervention completers versus noncompleters. When comparing study completers versus noncompleters, the only significant difference we found was the current professional help, with study noncompleters reporting use of medication, psychotherapy, or both more frequently than study completers (*P*<.001). Full details on the baseline characteristics of the total sample as well as of study completers versus noncompleters and intervention completers versus noncompleters can be found in Multimedia Appendix 1.

### Main RQs

#### RQ1: How Satisfied Are Participants With the New Online Intervention “GetStarted” and Its Platform?

##### Satisfaction

Satisfaction was assessed among participants who completed the posttest assessment (n=335). The average score on the CSQ-8 for the total sample was 23.48 (SD 3.23, scores ranging from 12 to 31). Intervention completers (n=175) had an average of 24.47 (SD 3.03), and intervention noncompleters (n=160) averaged 22.39 (SD 3.09). Intervention completers were significantly more satisfied than noncompleters (*t*_333_=6.212; *P*<.001; Cohen *d*=0.68).

##### Usability

Usability was assessed among participants who completed the posttest assessment (n=335). The average score on the SUS-10 for the total sample was 34.39 (SD 4.52, scores ranging from 16 to 40). Intervention completers (n=175) had an average of 35.51 (SD 3.68), while intervention noncompleters (n=160) averaged 33.17 (SD 5.01). The SUS-10 multiplies these scores by 2.5 to achieve a total score of 0-100. The total scores end up being 85.98 (total sample), 88.8 (completers), and 82.9 (noncompleters). Intervention completers reported significantly higher usability than noncompleters (*t*_333_=4.94; *P*<.001; Cohen *d*=0.54).

### RQ2: What Are the Adherence Rates of the New Intervention?

Adherence was calculated based on all participants who started the intervention (ie, completed at least 1 module; n=734). In this study, completing 4 main modules meant that the participant completed the intervention, which was done by 269 of 734 (36.65%) participants. This would indicate a dropout rate of 63.35% (n=465); however, participants who dropped out still partially adhered to the intervention. To capture this partial adherence, we calculated that participants completed an average of 2.97 out of 4 main modules, corresponding to 68.95% of the overall intervention.

Many participants also chose to do one or several optional modules. The most popular topics were “productivity” and “motivation boost,” which were completed by 26.7% (n=196) and 24.5% (n=180) of participants, respectively. The optional modules about “task management” and “time management” were completed by 19.5% and 14.9% of participants, respectively.

### Secondary RQs

#### RQ3: What Are the Differences Between Baseline to Posttest to Follow-Up for Procrastination, Depression, Perceived Stress Levels, and Quality of Life?

##### Procrastination, Mood, Stress, and Quality of Life Post Intervention

A total of 335 participants completed the posttest and were included in the analyses. We conducted a 2-tailed paired *t* test comparing the baseline and posttest for each outcome measure. Participants showed improvements in procrastination, depression, stress, and quality of life: IPS, PHQ-9, and PSS-10 scores all decreased significantly, while MHQoL scores increased significantly (all *P*<.001). The effect remains significant when correcting for multiple comparisons (type 1 error) using a Bonferroni-corrected α (*k*=4, adjusted α=0.0125). The standardized mean difference in procrastination scores was moderate to large (Cohen *d*=0.63). Standardized mean difference scores for depression, stress, and quality of life were small to moderate (Cohen *d*=0.35, 0.32, and 0.37, respectively). Full details can be found in Table 3.

**Table 3.** Secondary outcomes changes from baseline to posttest (n=335).

Secondary outcome	Baseline scores, mean (SD)	Posttest scores, mean (SD)	*t* test (*df*)	*P* value (2-tailed)	Cohen *d*	Cohen *d* 95% CI
IPS^a^	35.39 (4.31)	32.56 (5.34)	11.57 (334)	<.001	0.63	0.51-0.75
PHQ-9^b^	9.27 (4.7)	7.73 (4.57)	6.36 (334)	<.001	0.35	0.24-0.46
PSS-10^c^	20.79 (6.02)	19.02 (6.3)	5.64 (334)	<.001	0.31	0.2-0.42
MHQoL^d^	12.81 (3.02)	13.65 (2.93)	6.75 (334)	<.001	0.37	0.26-0.48

a IPS: Irrational Procrastination Scale.

b PHQ-9: 9-item Patient Health Questionnaire.

c PSS-10: 10-item Perceived Stress Scale.

d MHQoL: Mental Health Quality of Life Questionnaire.

##### Procrastination, Mood, Stress, and Quality of Life at Follow-Up

A series of mixed-effects repeated measures models was conducted to evaluate changes in procrastination, depression symptoms, perceived stress, and mental health quality of life over time (n=734). The models revealed significant changes in all 4 outcomes across the 3 time points (all *P*<.001). Procrastination scores decreased by 2.86 points from preintervention to postintervention and by 5.38 points at follow-up (*χ*²_2_=400.89). Depression symptoms were reduced by 1.62 points post intervention and by 2.58 points at follow-up (*χ*²_2_=109.04). Perceived stress scores declined by 1.96 points post intervention and by 3.51 points at follow-up (*χ*²_2_=111.89). Mental health quality of life improved over time, with increases of 0.87 points post intervention and 1.02 points at follow-up (*χ*²_2_=75.10). The models accounted for individual differences in baseline scores across all outcomes.

### RQ4: How Satisfied Are Participants with the E-Coaching Provided by Clinical Psychology Students?

E-coach satisfaction was assessed among participants who completed the posttest assessment (n=335). The average score on the WAI-I for the total sample was 45.26 (SD 7.72, scores ranging from 15 to 60). Participants who completed the intervention (n=175) reported higher satisfaction (mean 47.39, SD 6.91) than intervention noncompleters (n=160; mean 42.93, SD 0.62). The difference was significant (*t*_333_=5.52; *P*<.001; Cohen *d*=0.6).

## Discussion

### Principal Results

This study demonstrates promising outcomes for the feasibility and acceptability of “GetStarted,” an eHealth intervention targeting procrastination specifically designed for college students guided by psychology students. Participants reported high satisfaction with the intervention and found the system usable. Adherence rates were acceptable given the nature of the intervention (self-help), the topic (procrastination), and the target group (students). While most participants did not finish the intervention, on average, they still completed a large part of the intervention. Participants showed a moderate-to-large decrease in procrastination, a small-to-moderate decrease in depression and stress, and a small-to-moderate increase in quality of life between pre- and posttest. However, these results must be interpreted with caution given the lack of a control group in the current study design. Lastly, participants were sufficiently satisfied with the guidance of student e-coaches.

### Comparison With Prior Work

Our findings are in line with previous research on eHealth interventions among college students. Students report being satisfied with interventions for varying mental health problems such as stress, depression, and anxiety [54,74,75]. In this study, satisfaction scores were also found to be acceptable for the total sample and high for intervention completers. Internet interventions for mental health complaints are also generally found to have moderate-to-high usability [54,74,76,77]. Usability scores for the present intervention were high and corresponded with very good–to-excellent user experience [78].

Regarding intervention adherence, a meta-analysis reported dropout rates of up to 50.33% in guided and unguided internet interventions for college students [79]. More recent individual studies of guided internet interventions similar to ours reported dropout rates of 66.4% [74] and 48% [54]. In this study, 63.35% (n=288) of participants who started the intervention did not complete it. While this is on the high side, it is still in line with prior research. Additionally, on average, participants completed nearly 70% of the intervention and therefore were exposed to the larger part of the treatment. It is likely that participants benefited from this exposure to the treatment, even if they subsequently dropped out. We therefore deem our adherence rate on the low side, yet consider it acceptable in order to move forward with the “GetStarted” intervention.

This study also examined any changes to procrastination behavior, depression, stress, and quality of life in an exploratory fashion. While the absence of a control group means we cannot draw any conclusions on treatment effectiveness, we did see an improvement in all these areas. This is also in line with prior research, which has shown that internet interventions can be effective in treating different kinds of mental health problems in students, such as depression, anxiety, stress, and eating disorder symptoms [79] as well as procrastination [30-32]. The implication of our findings is 2-fold. First, this study provides preliminary indications that GetStarted may be a promising eHealth intervention to reduce procrastination behavior in college students, as reflected by a moderate-to-large effect size. Second, our findings tentatively suggest that targeting one specific problem such as procrastination can also potentially reduce other mental health complaints such as depression or stress, even if the intervention was not aimed at these problems specifically. However, it must be noted that spontaneous remission or regression to the norm cannot be ruled out in our study, given our single-arm design. Further research utilizing a control group is needed to draw conclusions on intervention effectiveness.

Lastly, we examined whether guidance by trained psychology students was acceptable. Our findings indicate sufficient satisfaction with the guidance, which is comparable to other guided, internet-based, or face-to-face interventions [74,80,81].

Considering these findings, the new internet intervention “GetStarted” targeting procrastination in college students appears very promising.

### Strengths and Limitations

A main strength of this study is the comprehensiveness of the assessment. When examining the acceptability and feasibility of new eHealth interventions, studies mainly focus on participant satisfaction and usability. While these were the main outcomes of this study, we also explored changes to procrastination and several other related mental health complaints. Given that procrastination is linked to mental health issues such as depression, social anxiety, stress, and low self-esteem [13,14,16,17], this study provides valuable insights into how treating procrastination may also impact mood, stress, and quality of life. Additionally, we employed posttest as well as follow-up measures. This allowed us to explore both short-term and longer term changes to these mental health complaints.

However, a major limitation of this study is that we cannot draw any conclusions about the effectiveness of the intervention on student mental health due to the lack of a control group. While this feasibility study was not designed to evaluate effectiveness, we did report pre-post change scores on secondary outcomes. While we found positive changes on all the measures, none of these can be directly attributed to the intervention given the lack of a control group. Regression to the mean or spontaneous remission offers alternative explanations for the improvements we found. Moreover, the observed effects may be inflated due to selective study attrition. Although a formal bias analysis falls outside the scope of this study, we acknowledge that the true change may be smaller than reported. Therefore, in future work, we plan to conduct a randomized controlled trial that includes a suitable comparison condition (eg, waitlist control, treatment-as-usual, or active placebo). This will allow us to more rigorously evaluate intervention effectiveness and account for nonspecific factors such as spontaneous remission and placebo effects.

A third limitation of this study regards the sample. During the recruitment process, all students of participating universities were offered access to the intervention. However, not all students are equally likely to be interested in or willing to sign up for the intervention, which means our findings are not directly generalizable to the total student population. For example, the majority of participants in this study were Dutch and female, which limits our understanding of intervention feasibility for other genders and nationalities. As of April 2025, 44.3% of university students in the Netherlands were male and 27.1% were international [82,83], compared to 25.2% (n=185) and 39.2% (n=288), respectively, in our sample. Our sample seems sufficiently diverse in terms of nationality; however, male students are underrepresented. A possible approach for future research is to first try to gain a deeper understanding of why male students in the Netherlands are less likely to make use of the intervention. Prior research has found that male students may perceive fewer benefits of and hold more stigma-related attitudes regarding mental health help-seeking [84]. Additional interviews with this target demographic could yield specific, actionable insights on how to increase the uptake of the “GetStarted” intervention. Additionally, targeted marketing efforts could be made. For example, colleges host many events for specific groups. These events can be a great opportunity to create awareness of the intervention among certain groups. Similarly, online marketing efforts such as social media posts could target certain demographics. A recommendation for future recruitment is to aim for a sample that is between 40% and 45% male participants. A more diverse study sample in further research on the intervention would greatly benefit the generalizability of future findings.

Lastly, the high attrition is a limitation in this study. Less than half of the participants who started the intervention completed the postmeasure, and about a third completed the 6-month follow-up. Though we found no differences in the baseline characteristics of study completers and noncompleters, we cannot rule out any unmeasured confounders. This results in a risk of attrition bias, meaning that “differences between people who leave a study and those that continue can be the reason for any observed effect and not the intervention itself” [85]. Future research should employ strategies to reduce attrition, such as calling participants as a reminder or offering a financial incentive, in order to avoid attrition bias. Both reminder-based approaches and financial incentives have been shown to improve retention in online intervention trials [86,87].

A strength of this study is our exploration of student e-coaches as the source of guidance. To the best of our knowledge, this study was the first to investigate the acceptability of guidance offered by trained psychology students. Prior research has shown that guided internet interventions are more effective than unguided treatment. However, mental health resources such as properly trained professionals are scarce. It is of paramount importance to find scalable and cost-effective alternatives to a licensed psychologist so that people in need can get timely mental health care. Clinical psychology students are required as well as eager to gain hands-on experience during their studies, which makes them a promising potential resource. This study shows that participants of our eHealth intervention are satisfied with the guidance offered by student coaches, and tapping into this more readily available resource means people in need can get more or quicker access to mental health support.

Finally, a last strength of this study is the scalability of our approach, starting with the guidance offered by e-coaches. In this study, e-coaches were involved as a part of their curriculum (ie, a “mini-internship”), meaning they did receive study credits but not financial compensation. This greatly adds to the scalability of our approach. While supervising more e-coaches does require slightly more resources in terms of work hours, this investment is negligible compared to the costs involved with utilizing mental health professionals.

The digital platform used to deliver the intervention was specifically developed with scalability in mind. The platform was created by the Caring Universities Project, which is affiliated with the World Health Organization’s WMH-ICS [51]. We currently offer access to 11 digital interventions, including “GetStarted,” and adding additional interventions incurs minimal additional cost. Participating institutions pay a membership fee in exchange for unlimited access to all interventions. Importantly, there is no cap on the number of students who can enroll. Although there are some ongoing maintenance costs associated with platform upkeep, these remain relatively modest, especially in light of the wide range of interventions and the large number of students that can be reached. Additionally, given the noncommercial nature of Caring Universities, the per-institution fee decreases as additional universities join the initiative, while platform costs remain stable. This model offers substantial potential for large-scale dissemination and implementation.

### Conclusions

The internet-based, student-guided intervention “GetStarted” targeting procrastination appears to be acceptable and feasible for college students in the Netherlands. The intervention could potentially also reduce complaints in the areas of procrastination, mood, and stress. Future research should determine possible intervention effectiveness using a control group, as well as explore strategies to address high study attrition.

## Supplementary material

10.2196/72896 Multimedia Appendix 1 Comparison of baseline characteristics.

10.2196/72896 Checklist 1 STROBE checklist.

## Abbreviations

CBT: cognitive behavioral therapy
CSQ-8: 8-item Client Satisfaction Questionnaire
IPS: Irrational Procrastination Scale
MHQoL: Mental Health Quality of Life Questionnaire
PHQ-9: 9-item Patient Health Questionnaire
PSS-10: 10-item Perceived Stress Scale
RQ: research question
STROBE: Strengthening the Reporting of Observational Studies in Epidemiology
SUS-10: 10-item System Usability Scale
WAI-I: Working Alliance Inventory for Guided Internet Interventions
WMH-ICS: World Mental Health International College Student Initiative
