# Supplementary material for: Feasibility of a Guided Web-Based Procrastination Intervention for College Students: Open Trial
Source: JMIR Form Res. 2025 Oct 16;9:e72896. doi: 10.2196/72896 (PMC12530647; doi:10.2196/72896)
Supplement: Multimedia Appendix 1 [file formative-v9-e72896-s001.docx]

Multimedia appendix for “Feasibility of a Guided Web-Based Procrastination Intervention for College Students: an Open Trial” (Manuscript ID: 72896)

**Baseline characteristics**

|  | Total (n=734) | Study |  |  | Intervention |  |  |
| --- | --- | --- | --- | --- | --- | --- | --- |
|  |  | Non-completers (n=399) | Completers (n=335) | *P* value | Non-completers (n=465) | Completers (n=269) | *P* value |
| **Age (years), mean (SD) ^a^** | **23.6 (4.15)** | **23.39 (3.73)** | **23.85 (4.59)** | **.14** | **23.52 (3.76)** | **23.74 (4.76)** | **.479** |
| **Gender, n (%) ^b^** |  |  |  | **.351** |  |  | **.146** |
| Female | 74 | 71.9 | 76.4 |  | 72.9 | 75.8 |  |
| Male | 25.2 | 27.3 | 22.7 |  | 25.8 | 24.2 |  |
| Other | 0.8 | 0.8 | 0.9 |  | 1.3 | 0 |  |
| **Nationality, n (%) ^b^** |  |  |  | **.433** |  |  | **.943** |
| European - Dutch | 60.8 | 61.4 | 60 |  | 60.6 | 61 |  |
| European - but not Dutch | 28.6 | 28.1 | 29.3 |  | 28.6 | 28.6 |  |
| North-American | 1.1 | 1.5 | 0.6 |  | 1.1 | 1.1 |  |
| South-American | 1.4 | 1.5 | 1.2 |  | 1.3 | 1.5 |  |
| Australian | 0.3 | 0.5 | 0 |  | 0.2 | 0.4 |  |
| Asian | 6.9 | 5.8 | 8.3 |  | 6.9 | 7.1 |  |
| African | 1 | 1.3 | 0.6 |  | 1.3 | 0.4 |  |
| **University, n (%) ^b^** |  |  |  | **.126** |  |  | **.949** |
| Maastricht | 12.4 | 11.8 | 13.1 |  | 12.3 | 12.6 |  |
| Erasmus | 5.2 | 5.3 | 5.1 |  | 4.9 | 5.6 |  |
| Inholland | 2.5 | 4 | 0.6 |  | 2.8 | 1.9 |  |
| Leiden | 23.6 | 22.8 | 24.5 |  | 23.2 | 24.2 |  |
| Utrecht | 31.5 | 31.1 | 31.9 |  | 30.8 | 32.7 |  |
| UvA | 9.9 | 9.3 | 10.7 |  | 10.3 | 9.3 |  |
| VU | 15 | 15.8 | 14 |  | 15.7 | 13.8 |  |
| **Education level, n (%) ^b^** |  |  |  | **.372** |  |  | **.84** |
| First year | 20 | 19.8 | 20.3 |  | 21.1 | 18.2 |  |
| Second year | 11.4 | 10.5 | 12.5 |  | 12 | 10.4 |  |
| Third year | 11.3 | 13.5 | 8.7 |  | 11 | 11.9 |  |
| Fourth year | 5.2 | 5.5 | 4.8 |  | 5.2 | 5.2 |  |
| Master’s degree student | 46.3 | 44.6 | 48.4 |  | 45.6 | 47.6 |  |
| PhD student | 5.7 | 6 | 5.4 |  | 5.2 | 6.7 |  |
| **Marital status, n (%) ^b^** |  |  |  | **.287** |  |  | **.677** |
| Single | 60.1 | 63.2 | 56.4 |  | 59.8 | 60.6 |  |
| In a relationship | 35.6 | 32.6 | 39.1 |  | 35.9 | 34.9 |  |
| Married | 3.1 | 3 | 3.3 |  | 3 | 3.3 |  |
| Divorced | 0.1 | 0 | 0.3 |  | 0 | 0.4 |  |
| Other | 1.1 | 1.3 | 0.9 |  | 1.3 | 0.7 |  |
| **Current professional help, n (%) ^b^** |  |  |  | **<.001** |  |  | **.114** |
| Medication | 5.7 | 6.8 | 4.5 |  | 6.2 | 4.8 |  |
| Psychotherapy or counseling | 7.5 | 11 | 3.3 |  | 8.4 | 5.9 |  |
| Both | 3 | 2.3 | 3.9 |  | 3.9 | 1.5 |  |
| None | 83.8 | 79.7 | 88.4 |  | 81.5 | 87.7 |  |
| **Baseline clinical characteristics, mean (SD) ^a^** |  |  |  |  |  |  |  |
| IPS | 35.52 (4.37) | 35.64 (4.43) | 35.39 (4.31) | .443 | 35.54 (4.25) | 35.49 (4.59) | .879 |
| PHQ-9 | 9.37 (4.99) | 9.44 (5.23) | 9.27 (4.7) | .642 | 9.48 (5) | 9.16 (4.98) | .406 |
| PSS-10 | 21.13 (6.24) | 21.42 (6.41) | 20.79 (6.02) | .177 | 21.3 (6.2) | 20.84 (6.31) | .344 |
| MHQoL | 12.72 (2.98) | 12.65 (2.95) | 12.81 (3.02) | .454 | 12.68 (2.92) | 12.8 (3.09) | .594 |

a2-tailed Independent sample *t* test.
bChi-square test
