# Supplementary material for: Feasibility of a Guided Web-Based Procrastination Intervention for College Students: Open Trial
Source: JMIR Form Res. 2025 Oct 16;9:e72896. doi: 10.2196/72896 (PMC12530647; doi:10.2196/72896)
Supplement: Checklist 1 [file formative-v9-e72896-s002.pdf]

STROBE Statement—checklist of items that should be included in reports of observational studies

| | Item No. | Recommendation | Page No. | Relevant text from manuscript |
|---|---|---|---|---|
| **Title and abstract** | 1 | (*a*) Indicate the study's design with a commonly used term in the title or the abstract | 1 | **Feasibility of a Guided Web-Based Procrastination Intervention for College Students: an Open Trial** |
| | | (*b*) Provide in the abstract an informative and balanced summary of what was done and what was found | 1 | **Objective:** This study aims to examine the feasibility and acceptability of a new e-health intervention targeting procrastination for college students ('GetStarted') with guidance by student e-coaches. This CBT-based intervention was designed specifically for and together with the target demographic of students studying in the Netherlands. Guidance was offered by trained Clinical Psychology students in the form of written motivational, supportive messages. **Methods:** We conducted a single-arm study. Primary outcomes are satisfaction (CSQ-8), usability, (SUS-10) and adherence (completion rate). Secondary outcomes are changes to |

procrastination (IPS), depression (PHQ-9), stress (PSS-10), quality of life (MHQoL) and e-coaching satisfaction (WAI-I).

**Results:**

Of 734 participants that started the intervention, 335 (45.6%) completed the post-test. Students report being satisfied with the intervention (CSQ-8 M = 23.48; SD = 3.23) and find it very usable (SUS-10 M = 34.39; SD = 4.52). Regarding adherence, participants completed 68.95% of the intervention on average, while 36.65% participants completed the full intervention. Participants showed a significant decrease in procrastination (IPS M = 35.39 to 32.56, Cohen's d = .63), depression (PHQ-9 M = 9.27 to 7.73, Cohen's d = .35) and stress (PSS-10 M = 20.79 to 19.02, Cohen's d = .31) as well as an increase in quality of life (MHQoL M = 12.81 to 13.65, Cohen's d = .37) from baseline to post-test to follow-up. Participants reported a moderate

| | | | | |
|---|---|---|---|---|
| | | | | to strong alliance with their e-coach (WAI-I M = 45.26; SD = 7.72). |
| **Introduction** | | | | |
| Background/rationale | 2 | Explain the scientific background and rationale for the investigation being reported | 2-3 | It is therefore important to find and implement effective treatments that could help students understand and decrease their procrastination behaviors. Meta-analytical evidence shows that effective treatments for procrastination exist [20], and that cognitive behavior therapy (CBT) was most effective in reducing procrastination in comparison with self-regulation training, strengths training, and acceptance-based behavior therapy [21].<br><br>In recent years, e-health interventions have gained popularity and proved well-suited to college students' dynamic lifestyles and diverse challenges [27–29]. From accessibility, convenience, cost-effectiveness and anonymity to the possibilities of |

personalization, promotion of health literacy, feedback mechanisms and cultural and linguistic inclusivity, e-health interventions address many barriers to treatments commonly faced by students [27]. In line with this, some studies have shown that e-health interventions effectively reduce procrastination among college students [30–32]. Compared to face-to-face, chat-based, and in-person group therapy, e-health treatment has yielded comparable results in improving procrastination behaviors [33,34].

We developed a new online intervention targeting procrastination (‘GetStarted’) for and in collaboration with students studying in the Netherlands, addressing the lack of existing interventions specifically designed for this group. This allowed us to ensure that the contents of the intervention were relevant and appealing to this target demographic. The intervention

| | | | | |
|---|---|---|---|---|
| | | | | was based on cognitive behavior therapy (CBT), given meta-analytical evidence that this is the most effective type of intervention [21]. Specifically, the intervention utilized cognitive restructuring, which is a key mechanism of change in CBT [50].<br><br>Due to its novel nature, the intervention has not yet been examined in the scientific literature. Therefore, the current study evaluates the feasibility and accessibility of a newly developed online intervention guided by psychology student e-coaches to reduce procrastination among college students. |
| Objectives | 3 | State specific objectives, including any prespecified hypotheses | 3 | Main RQs:<br><br>RQ1: How satisfied are participants with the new online intervention 'GetStarted' and its platform?<br>RQ2: What are the adherence rates of the new intervention?<br><br>Secondary RQs: |

| | | | | |
|---|---|---|---|---|
| | | | | RQ3: What are the differences between baseline to post-test to follow up for procrastination, depression, perceived stress levels and quality of life?<br>RQ4: How satisfied are participants with the e-coaching provided by clinical psychology students? |
| **Methods** | | | | |
| Study design | 4 | Present key elements of study design early in the paper | 3-4 | A single-arm open trial design was used to assess feasibility and acceptability of a new e-health intervention aimed at reducing procrastination among college students, which are the primary aims at this stage. This time- and cost-efficient approach allowed for initial insights into engagement and implementation, before proceeding to a randomized controlled trial to better examine effectiveness. In this single arm within-group design, assessments were administered at baseline (t0), post intervention (t2) and follow-up (t2) occurring at 4 weeks and 6 months post baseline respectively. |

| | | | | |
|---|---|---|---|---|
| Setting | 5 | Describe the setting, locations, and relevant dates, including periods of recruitment, exposure, follow-up, and data collection | 4 | Participants were students enrolled in any one of the seven Dutch universities within the Caring Universities consortium. Recruitment took place in three ways from January 2021 to December 2023. First, through an annual online mental health survey of the WMH-ICS. Participants who scored above 28 points on the Irrational Procrastination Scale (IPS) in the survey were invited to participate in the intervention. Second, through social media marketing as well as on-campus marketing via the distribution of posters, flyers, and other promotional materials. Third, college staff members such as student psychologists, study advisors, and lecturers could recommend the intervention to students that might benefit from it. |
| Participants | 6 | (*a*) *Cohort study*—Give the eligibility criteria, and the sources and methods of selection of participants. Describe methods of follow-up<br>*Case-control study*—Give the eligibility criteria, and the sources and methods of case ascertainment and control selection. Give the rationale for the choice of cases and controls<br>*Cross-sectional study*—Give the eligibility criteria, and the sources and methods of selection of participants | 4 | **Eligibility Criteria**<br>Participants were eligible if they were 1) aged 16 years or older, 2) enrolled as a student in one of the participating universities, 3) reported no active suicidal ideation and 4) provided |

| | | | | |
|---|---|---|---|---|
| | | | | informed consent. |
| | | (*b*) *Cohort study*—For matched studies, give matching criteria and number of exposed and unexposed<br>*Case-control study*—For matched studies, give matching criteria and the number of controls per case | | |
| Variables | 7 | Clearly define all outcomes, exposures, predictors, potential confounders, and effect modifiers. Give diagnostic criteria, if applicable | 6-7 | **Assessment measures**<br>***Primary outcomes***<br>**Satisfaction with the intervention**<br>The Client Satisfaction Questionnaire (CSQ-8) was used to evaluate participant's satisfaction with the intervention [58]. It consists of eight questions on a 4-point Likert scale (sum score 8-32) and a higher score indicates greater satisfaction. The CSQ-8 has shown high reliability and validity for online interventions [59].<br><br>**Usability**<br>Usability of the intervention was assessed using the System Usability Scale (SUS-10) [60]. It consists of 10 questions on a 5-point Likert scale (sum score 0-40). Total scores are then multiplied by 2.5 to achieve a total score of 0 to 100, where a |

higher score indicates greater usability. The SUS-10 has shown good psychometric properties (reliability and validity) [61].

**Adherence**

Adherence is "the degree to which the user followed the program as it was designed" [62]. In this study, modules 1-4 were considered core module as they contained the CBT elements. Therefore, participants who completed these four core modules were considered completers of the intervention. Additionally, average adherence across the total sample was calculated by dividing the number of core modules completed by the total number of core modules in the program and multiplying this by 100. The resulting percentage indicates adherence rate. Participants who did not complete a single module were excluded from the analyses as they did not start the treatment.

***Secondary outcomes***

**Procrastination tendencies**

Procrastination behavior was measured using the Irrational Procrastination Scale (IPS) [63,64]. It consists of 9 questions on a 5-point Likert scale (sum score 9-45), and a higher score indicates more procrastination behavior. The IPS showed good internal consistency of a = 0.91 [63], a high level of reliability (rpm = 0.58 - 0.74), and good content, structural, and substantive validity [65].

**Depressive symptoms**

The PHQ-9 was used to measure depression [66]. It comprises 9 questions on a 4-point Likert scale (sum score 0-27) and a higher score indicates a higher level of depression. The PHQ-9 showed high sensitivity (0.71 - 0.84), specificity (0.90 – 0.97), internal consistency (Cronbach's $\alpha$ = 0.86 – 0.89), test-retest reliability (r = 0.84) and validity [66,67].

**Perceived stress**

The Perceived Stress Scale

(PSS-10) was used to measure stress [68,69]. It comprises 10 questions on a 5-point Likert scale (sum score 0-40), where a higher score indicates higher perceived stress. The PSS-10 showed high validity, internal consistency (Cronbach's α= 0.74 - 0.91) and test-retest reliability (r = 0.74 – 0.88) [69,70].

**Quality of life**

The Mental Health Quality of Life questionnaire (MHQoL) was used to assess quality of life [71]. It comprises 7 questions about different dimensions of life (e.g. mood, relationships) on a 4-point Likert scale (sum score 0-21), where a higher score indicates better quality of life. The MHQoL showed good validity, test-retest reliability (r = 0.85) and internal consistency (Cronbach's α= 0.85) [71].

**E-coach evaluation**

Participant's satisfaction with the e-coach was assessed using the Working Alliance Inventory for guided internet interventions

(WAI-I) [72]. It comprises 12 questions on a 5-point Likert scale (sum score 12-60), where higher scores indicate higher satisfaction. A score of 36 or higher indicates sufficient satisfaction, where a score of 48 to 60 means high to very high satisfaction with the e-coach. The psychometric characteristics of the WAI-I yielded adequate results [72].

**Sociodemographic information**

The following sociodemographic information was collected: age, gender, marital status, nationality, attending university, faculty, education level (Bachelor, Master or Ph.D.), and whether psychotherapy and/or medication were received. This information was collected firstly to gain insight into the characteristics of individuals that are interested in the intervention when offered access to it in a real-life setting. Second, we wanted to ensure that there were no major

| | | | | |
|---|---|---|---|---|
| | | | | differences between participants that did and did not complete the study to account for high attrition rates. |
| Data sources/ measurement | 8* | For each variable of interest, give sources of data and details of methods of assessment (measurement). Describe comparability of assessment methods if there is more than one group | 6-7 | **Assessment measures** **Primary outcomes** **Satisfaction with the intervention** The Client Satisfaction Questionnaire (CSQ-8) was used to evaluate participant's satisfaction with the intervention [58]. It consists of eight questions on a 4-point Likert scale (sum score 8-32) and a higher score indicates greater satisfaction. The CSQ-8 has shown high reliability and validity for online interventions [59].<br><br>**Usability** Usability of the intervention was assessed using the System Usability Scale (SUS-10) [60]. It consists of 10 questions on a 5-point Likert scale (sum score 0-40). Total scores are then multiplied by 2.5 to achieve a total score of 0 to 100, where a higher score indicates greater |

usability. The SUS-10 has shown good psychometric properties (reliability and validity) [61].

**Adherence**

Adherence is "the degree to which the user followed the program as it was designed" [62]. In this study, modules 1-4 were considered core module as they contained the CBT elements. Therefore, participants who completed these four core modules were considered completers of the intervention. Additionally, average adherence across the total sample was calculated by dividing the number of core modules completed by the total number of core modules in the program and multiplying this by 100. The resulting percentage indicates adherence rate. Participants who did not complete a single module were excluded from the analyses as they did not start the treatment.

**62. Secondary outcomes**

**Procrastination tendencies**

Procrastination behavior was

measured using the Irrational Procrastination Scale (IPS) [63,64]. It consists of 9 questions on a 5-point Likert scale (sum score 9-45), and a higher score indicates more procrastination behavior. The IPS showed good internal consistency of a = 0.91 [63], a high level of reliability (rpm = 0.58 - 0.74), and good content, structural, and substantive validity [65].

**Depressive symptoms**

The PHQ-9 was used to measure depression [66]. It comprises 9 questions on a 4-point Likert scale (sum score 0-27) and a higher score indicates a higher level of depression. The PHQ-9 showed high sensitivity (0.71 - 0.84), specificity (0.90 – 0.97), internal consistency (Cronbach's α = 0.86 – 0.89), test-retest reliability (r = 0.84) and validity [66,67].

**Perceived stress**

The Perceived Stress Scale (PSS-10) was used to measure

stress [68,69]. It comprises 10 questions on a 5-point Likert scale (sum score 0-40), where a higher score indicates higher perceived stress. The PSS-10 showed high validity, internal consistency (Cronbach's α= 0.74 - 0.91) and test-retest reliability (r = 0.74 – 0.88) [69,70].

**Quality of life**

The Mental Health Quality of Life questionnaire (MHQoL) was used to assess quality of life [71]. It comprises 7 questions about different dimensions of life (e.g. mood, relationships) on a 4-point Likert scale (sum score 0-21), where a higher score indicates better quality of life. The MHQoL showed good validity, test-retest reliability (r = 0.85) and internal consistency (Cronbach's α= 0.85) [71].

**E-coach evaluation**

Participant's satisfaction with the e-coach was assessed using the Working Alliance Inventory for guided internet interventions (WAI-I) [72]. It comprises 12

questions on a 5-point Likert scale (sum score 12-60), where higher scores indicate higher satisfaction. A score of 36 or higher indicates sufficient satisfaction, where a score of 48 to 60 means high to very high satisfaction with the e-coach. The psychometric characteristics of the WAI-I yielded adequate results [72].

**Sociodemographic information**

The following sociodemographic information was collected: age, gender, marital status, nationality, attending university, faculty, education level (Bachelor, Master or Ph.D.), and whether psychotherapy and/or medication were received. This information was collected firstly to gain insight into the characteristics of individuals that are interested in the intervention when offered access to it in a real-life setting. Second, we wanted to ensure that there were no major differences between participants

| | | | | |
|---|---|---|---|---|
| | | | | that did and did not complete the study to account for high attrition rates. |
| Bias | 9 | Describe any efforts to address potential sources of bias | 7, 13-14 | Firstly, we examined the baseline characteristics of the whole sample, study completers vs. non-completers (i.e., participants who did vs. did not complete the post-test assessment), and intervention completers vs. non-completers (i.e., participants who did vs. did not complete at least 4 main modules). Potential differences in baseline characteristics between these groups were examined using Chi-squared tests and independent sample t-tests.<br><br>Though we found no differences in the baseline characteristics of study completers and non-completers, we cannot rule out any unmeasured confounders. This results in a risk of attrition bias, meaning that 'differences between people who leave a study and those that continue can be the reason for any observed effect and not the |

| | | | | |
|---|---|---|---|---|
| | | | | intervention itself' [85]. Future research should employ strategies to reduce attrition, such as calling participants as a reminder or offering a financial incentive, in order to avoid attrition bias. |
| Study size | 10 | Explain how the study size was arrived at | 4 | **Sample size**<br>There is no standardized method for determining the sample size of an open feasibility study and previous research has recommended 12 [52] to 35 or more participants [53]. Drawing on these guidelines and comparable studies [54,55], we estimated that 50 participants would be sufficient to address our primary objectives. However, the present intervention was available to the general student population for several years and we expected that more than 50 participants would sign up. For quality assurance, we also conducted a power analysis and aiming for a conservative 2-tailed calculation with a small effect size (0.2), our statistical analyses (estimating differences between two dependent means) would |

| | | | | |
|---|---|---|---|---|
| | | | | require a total sample size of 199 participants [56]. In the current paper, we included all participants who had used the intervention and completed the post-test at the time of data analysis to be able to draw firmer conclusions. |

Continued on next page

| | | | | |
|---|---|---|---|---|
| Quantitative variables | 11 | Explain how quantitative variables were handled in the analyses. If applicable, describe which groupings were chosen and why | 4, 8 | In the current paper, we included all participants who had used the intervention and completed the post-test at the time of data analysis to be able to draw firmer conclusions.<br><br>For our primary outcomes of client satisfaction (CSQ-8) and usability (SUS-10), we conducted a complete case analyses (CCA) and analyzed only study completers. We calculated descriptive statistics for the whole sample and examined intervention completers vs. non-completers as an additional sensitivity analysis. For the primary outcome of adherence, we calculated how much of the program was completed on average, as well as the percentage of participants who completed the intervention vs. did not complete the intervention.<br><br>For our secondary outcomes, we examined whether there was a statistical difference from baseline to post-test in reported procrastination behavior (IPS), depression (PHQ-9), stress (PSS-10) and quality of life (MHQoL) for |

| | | | | |
|---|---|---|---|---|
| | | | | intervention completers. We conducted two-tailed paired t-tests using a significance level α = 0.05 to assess these changes. To interpret the effect size, we calculated the Cohen`s d, interpreting the benchmarks of 0.2, 0.5, and 0.8 as small, moderate, and large, respectively [73]. |
| Statistical methods | 12 | (*a*) Describe all statistical methods, including those used to control for confounding | 7-8 | IBM SPSS version 27 was used for the data analyses.<br>Firstly, we examined the baseline characteristics of the whole sample, study completers vs. non-completers (i.e., participants who did vs. did not complete the post-test assessment), and intervention completers vs. non-completers (i.e., participants who did vs. did not complete at least 4 main modules). Potential differences in baseline characteristics between these groups were examined using Chi-squared tests and independent sample t-tests.<br><br>For our primary outcomes of client satisfaction (CSQ-8) and usability (SUS-10), we conducted a complete case analyses (CCA) and analyzed only study completers. We |

| | | | |
|---|---|---|---|
| | | | calculated descriptive statistics for the whole sample and examined intervention completers vs. non-completers as an additional sensitivity analysis. For the primary outcome of adherence, we calculated how much of the program was completed on average, as well as the percentage of participants who completed the intervention vs. did not complete the intervention.<br><br>For our secondary outcomes, we examined whether there was a statistical difference from baseline to post-test in reported procrastination behavior (IPS), depression (PHQ-9), stress (PSS-10) and quality of life (MHQoL) for intervention completers. We conducted two-tailed paired t-tests using a significance level $\alpha = 0.05$ to assess these changes. To interpret the effect size, we calculated the Cohen`s d, interpreting the benchmarks of 0.2, 0.5, and 0.8 as small, moderate, and large, respectively [73]. |
| | (*b*) Describe any methods used to examine subgroups and interactions | 8 | For our primary outcomes of client satisfaction (CSQ-8) and usability (SUS-10), we conducted a complete |

| | | | |
|---|---|---|---|
| | | | case analyses (CCA) and analyzed only study completers. We calculated descriptive statistics for the whole sample and examined intervention completers vs. non-completers as an additional sensitivity analysis. For the primary outcome of adherence, we calculated how much of the program was completed on average, as well as the percentage of participants who completed the intervention vs. did not complete the intervention. |
| | (*c*) Explain how missing data were addressed | 8 | For our primary outcomes of client satisfaction (CSQ-8) and usability (SUS-10), we conducted a complete case analyses (CCA) and analyzed only study completers.<br><br>For our secondary outcomes which were of an exploratory nature, we conducted a complete case analyses (CCA) and examined whether there was a statistical difference from baseline to post-test in reported procrastination behavior (IPS), depression (PHQ-9), stress (PSS-10) and quality of life (MHQoL). We conducted two-tailed paired t-tests using a significance level $\alpha$ = 0.05 to assess these changes. To |

| | | | |
|---|---|---|---|
| | | | interpret the effect size, we calculated the Cohen`s d, interpreting the benchmarks of 0.2, 0.5, and 0.8 as small, moderate, and large, respectively [73]. |
| | (*d*) *Cohort study*—If applicable, explain how loss to follow-up was addressed<br>*Case-control study*—If applicable, explain how matching of cases and controls was addressed<br>*Cross-sectional study*—If applicable, describe analytical methods taking account of sampling strategy | 8 | For our primary outcomes of client satisfaction (CSQ-8) and usability (SUS-10), we conducted a complete case analyses (CCA) and analyzed only study completers. We calculated descriptive statistics for the whole sample and examined intervention completers vs. non-completers as an additional sensitivity analysis. For the primary outcome of adherence, we calculated how much of the program was completed on average, as well as the percentage of participants who completed the intervention vs. did not complete the intervention.<br><br>For our secondary outcomes which were of an exploratory nature, we conducted a complete case analyses (CCA) and examined whether there was a statistical difference from baseline to post-test in reported procrastination behavior (IPS), depression (PHQ-9), stress (PSS- |

| | | | | |
|---|---|---|---|---|
| | | | | 10) and quality of life (MHQoL). We conducted two-tailed paired t-tests using a significance level α = 0.05 to assess these changes. To interpret the effect size, we calculated the Cohen`s d, interpreting the benchmarks of 0.2, 0.5, and 0.8 as small, moderate, and large, respectively [73]. |
| | | (*e*) Describe any sensitivity analyses | 8 | We calculated descriptive statistics for the whole sample and examined intervention completers vs. non-completers as an additional sensitivity analysis. |
| **Results** | | | | |
| Participants | 13* | (a) Report numbers of individuals at each stage of study—eg numbers potentially eligible, examined for eligibility, confirmed eligible, included in the study, completing follow-up, and analysed | 8 | A total of 1746 students were assessed for eligibility for the intervention between January 2021 and May 2023. Of these, 657 participants did not receive access to the intervention because they dropped out during the baseline assessment (n=204), dropped out during account creation (n=359), or were excluded due to active suicidal ideation (n=94).<br><br>1089 participants were granted access to the intervention. Of these, 355 (32,6%) did not start the intervention. The reasons for not |

| | | | |
|---|---|---|---|
| | | | accessing the intervention were not activating their account via a link they received per email (n=121) or completing zero modules within the intervention and therefore not starting the treatment (n=234). There were no baseline differences between those who started the intervention and those who did not.<br><br>A total of 734 participants started the intervention. Our analyses on adherence were conducted based on this sample. Of these 734 participants, 335 completed the post-test (i.e. study completers) and were included in the satisfaction and clinical outcome analyses. Details on the participant flow can be found in figure 1. |
| | (b) Give reasons for non-participation at each stage | 8 | A total of 1746 students were assessed for eligibility for the intervention between January 2021 and May 2023. Of these, 657 participants did not receive access to the intervention because they dropped out during the baseline assessment (n=204), dropped out during account creation (n=359), or were excluded due to active suicidal ideation (n=94). |

| | | | | |
|---|---|---|---|---|
| | | | | 1089 participants were granted access to the intervention. Of these, 355 (32,6%) did not start the intervention. The reasons for not accessing the intervention were not activating their account via a link they received per email (n=121) or completing zero modules within the intervention and therefore not starting the treatment (n=234). There were no baseline differences between those who started the intervention and those who did not.<br><br>A total of 734 participants started the intervention. Our analyses on adherence were conducted based on this sample. Of these 734 participants, 335 completed the post-test (i.e. study completers) and were included in the satisfaction and clinical outcome analyses. Details on the participant flow can be found in figure 1. |
| | | (c) Consider use of a flow diagram | 9 | Flow diagram is an image. |
| Descriptive data | 14* | (a) Give characteristics of study participants (eg demographic, clinical, social) and information on exposures and potential confounders | 8 | The average age of the total sample (n = 734) was 23.6 (*SD* = 4.15) and most participants were female (74%). Most were from the Netherlands (60.8%) or another country in Europe (28.6%). Almost half of the participants were |

| | | |
|---|---|---|
| | | currently doing their master's (46.3%). A vast majority of participants (83.8%) were currently receiving no medication or psychotherapy. We compared baseline characteristics of intervention completers and non-completers as well as study completers and non-completers. Age, gender, nationality, university, education level, marital status, current professional help and baseline clinical characteristics (procrastination, depression, stress, quality of life) were compared. We found no significant differences between intervention completers vs. non-completers. When comparing study completers vs. non-completers, the only significant difference we found was the current professional help, with study non-completers reporting use of medication, psychotherapy or both more frequently than study completers ($p < .001$). Full details on the baseline characteristics of the total sample as well as of study completers vs noncompleters and intervention completers vs non-completers can be found in the multimedia appendix. |

| | | | | |
|---|---|---|---|---|
| | | (b) Indicate number of participants with missing data for each variable of interest | 8 | A total of 1746 students were assessed for eligibility for the intervention between January 2021 and May 2023. Of these, 657 participants did not receive access to the intervention because they dropped out during the baseline assessment (n=204), dropped out during account creation (n=359), or were excluded due to active suicidal ideation (n=94).<br><br>1089 participants were granted access to the intervention. Of these, 355 (32,6%) did not start the intervention. The reasons for not accessing the intervention were not activating their account via a link they received per email (n=121) or completing zero modules within the intervention and therefore not starting the treatment (n=234). |
| | | (c) *Cohort study*—Summarise follow-up time (eg, average and total amount) | 7 | Table showing assessment points |
| Outcome data | 15* | *Cohort study*—Report numbers of outcome events or summary measures over time | 10 | ***Satisfaction***<br>Satisfaction was assessed among participants who completed the post-test assessment (n = 335). The average score on the CSQ-8 for the total sample was 23.48 (SD = 3.23, scores ranging 12-31). Intervention completers (n = 175) had an average of 24.47 (SD 3.03) and |

| | | | | |
|---|---|---|---|---|
| | | | | intervention non-completers (n = 160) averaged 22.39 (SD = 3.09). Intervention completers were significantly more satisfied than non-completers (t(333) = 6.212, *P*<.001, Cohen's d = .68).<br>***Usability***<br>Usability was assessed among participants who completed the post-test assessment (n = 335).The average score on the SUS-10 for the total sample was 34.39 (SD = 4.52, scores ranging 16-40). Intervention completers (n = 175) had an average of 35.51 (SD = 3.68), while intervention non-completers (n = 160) averaged 33.17 (SD = 5.01). The SUS-10 multiplies these scores by 2.5 to achieve a total score of 0 to 100. The total scores end up being 85.98 (total sample), 88.8 (completers) and 82.9 (non-completers). Intervention completers reported significantly higher usability than non-completers (t(333) = 4.94, *P*<.001, Cohen's d = .54). |
| | | *Case-control study*—Report numbers in each exposure category, or summary measures of exposure | | |
| | | *Cross-sectional study*—Report numbers of outcome events or summary measures | | |
| Main results | 16 | (*a*) Give unadjusted estimates and, if applicable, confounder-adjusted estimates and their precision (eg, 95% confidence interval). Make clear which confounders were adjusted for and why they were | 10-11 | *RQ1: How satisfied are participants with the new online* |

| | | |
|---|---|---|
| included | | *intervention 'GetStarted' and its platform?*<br>***Satisfaction***<br>Satisfaction was assessed among participants who completed the post-test assessment (n = 335). The average score on the CSQ-8 for the total sample was 23.48 (SD = 3.23, scores ranging 12-31). Intervention completers (n = 175) had an average of 24.47 (SD 3.03) and intervention non-completers (n = 160) averaged 22.39 (SD = 3.09). Intervention completers were significantly more satisfied than non-completers (t(333) = 6.212, *P*<.001, Cohen's d = .68).<br>***Usability***<br>Usability was assessed among participants who completed the post-test assessment (n = 335).The average score on the SUS-10 for the total sample was 34.39 (SD = 4.52, scores ranging 16-40). Intervention completers (n = 175) had an average of 35.51 (SD = 3.68), while intervention non-completers (n = 160) averaged 33.17 (SD = 5.01). The SUS-10 multiplies these scores by 2.5 to achieve a total score of 0 to 100. The total scores end up being 85.98 (total sample), 88.8 |

(completers) and 82.9 (non-completers). Intervention completers reported significantly higher usability than non-completers (t(333) = 4.94, $P$<.001, Cohen's d = .54).

*RQ2: What are the adherence rates of the new intervention?*

Adherence was calculated based on all participants that started the intervention (i.e., completed at least one module) (n = 734). In this study, completing four main modules meant that the participant completed the intervention, which was done by 269 out of 734 participants (36.65%). This would indicate a drop-out rate of 63.35%, however, participants that dropped out still partially adhered to the intervention. To capture this partial adherence, we calculated that participants completed an average of 2,97 out of 4 main modules, corresponding to 68.95% of the overall intervention.

Many participants also chose to do one or several optional modules. The most popular topics were

‘productivity’ and ‘motivation boost’, which were completed by 26.7% and 24.5% of participants respectively. The optional modules about ‘task management’ and ‘time management’ were completed by 19.5% and 14.9% of participants respectively.

Secondary RQs:

*RQ3: What are the differences between baseline to post-test to follow up for procrastination, depression, perceived stress levels and quality of life?*

***Procrastination, mood, stress and quality of life at post intervention***

A total of 335 participants completed the post-test and were included in the analyses. We conducted a two-tailed paired t-test comparing the baseline and post-test for each outcome measure. Participants showed improvements in procrastination, depression, stress and quality of life: IPS, PHQ-9 and PSS-10 scores all decreased significantly while MHQoL scores increased significantly (all $P<.001$). The effect remains significant when correcting for multiple comparisons

(type I error) using a Bonferroni-corrected alpha (k = 4, adjusted α = 0.0125). The standardized mean difference in procrastination scores was moderate to large (Cohen`s d = .63). Standardized mean difference scores for depression, stress and quality of life were small to moderate (Cohen`s d = .35, .32 and .37 respectively). Full details can be found in Table 3.

***Procrastination, mood, stress and quality of life at follow-up***

A series of mixed-effects repeated measures models were conducted to evaluate changes in procrastination, depression symptoms, perceived stress, and mental health quality of life over time (n=734). The models revealed significant changes in all four outcomes across the three time points (all $P<0.001$).
Procrastination scores decreased by 2.86 points from pre-intervention to post-intervention and by 5.38 points at follow-up ($\chi^2(2) = 400.89$). Depression symptoms were reduced by 1.62 points post-intervention and by 2.58 points at follow-up ($\chi^2(2) = 109.04$). Perceived stress scores declined by 1.96 points post-

| | | |
|---|---|---|
| | | intervention and by 3.51 points at follow-up ($\chi^2(2) = 111.89$). Mental health quality of life improved over time, with increases of 0.87 points post-intervention and 1.02 points at follow-up ($\chi^2(2) = 75.10$). The models accounted for individual differences in baseline scores across all outcomes.<br><br>*RQ4: How satisfied are participants with the e-coaching provided by clinical psychology students?*<br><br>E-coach satisfaction was assessed among participants who completed the post-test assessment (n = 335). The average score on the WAI-I for the total sample was 45.26 (SD = 7.72, scores ranging 15-60). Participants who completed the intervention (n = 175) reported higher satisfaction (M = 47.39, SD = 6.91) than intervention non-completers (n = 160, M = 42.93, SD = .62). The difference was significant (t(333) = 5.52, *P*<.001, Cohen's d = .6). |
| | (*b*) Report category boundaries when continuous variables were categorized | N/A |
| | (*c*) If relevant, consider translating estimates of relative risk into absolute risk for a meaningful time period | N/A |

Continued on next page

| | | | | |
|---|---|---|---|---|
| Other analyses | 17 | Report other analyses done—eg analyses of subgroups and interactions, and sensitivity analyses | 10-11 | All analyses are mentioned above. |
| **Discussion** | | | | |
| Key results | 18 | Summarise key results with reference to study objectives | 11-12 | **Principal Results**<br>This study demonstrates promising outcomes for the feasibility and acceptability of "GetStarted"; an e-health intervention targeting procrastination specifically designed for college students guided by psychology students. Participants reported high satisfaction with the intervention and found the system usable. Adherence rates were acceptable given the nature of the intervention (self-help), the topic (procrastination) and the target group (students). While most participants did not finish the intervention, on average they still completed a large part of the intervention. Participants showed a moderate to large decrease in procrastination, a small to moderate decrease in depression and stress, and a small to moderate increase in quality of life between pre- and posttest. However, these results must be interpreted with caution given the lack of control group in the current study design. Lastly, |

participants were sufficiently satisfied with the guidance of student e-coaches.

**Comparison with Prior Work**

Our findings are in line with previous research into e-health interventions among college students. Students report being satisfied with interventions for varying mental health problems such as stress, depression and anxiety [54,74,75]. In the present study, satisfaction scores were also found to be acceptable for the total sample and high for intervention completers. Internet interventions for mental health complaints are also generally found to have moderate to high usability [54,74,76,77]. Usability scores for the present intervention were high and corresponded with very good to excellent user experience [78].

Regarding intervention adherence, a meta-analysis reported dropout rates of up to 50.33% in guided and unguided internet interventions for college students [79]. More recent individual studies of guided internet interventions similar to ours reported dropout rates of 66,4%

[74] and 48% [54]. In the present study, 63.35% of participants who started the intervention did not complete it. While this is on the high side, it is still in line with prior research. Additionally, on average participants completed nearly 70% of the intervention and therefore were exposed to the larger part of the treatment. It is likely that participants benefited from this exposure to the treatment, even if they subsequently dropped out. We therefore deem our adherence rate on the low side, yet consider it acceptable in order to move forward with the 'GetStarted' intervention.

This study also examined any changes to procrastination behavior, depression, stress and quality of life in an exploratory fashion. While the absence of a control group means we cannot draw any conclusions on treatment effectiveness, we did see an improvement in all these areas. This is also in line with prior research, which has shown that internet interventions can be effective in treating different kinds of mental health problems in students, such as depression,

anxiety, stress, and eating disorder symptoms [79] as well as procrastination [30–32]. The implication of our findings is twofold. Firstly, this study provides preliminary indications that GetStarted may be a promising e-health intervention to reduce procrastination behavior in college students, as reflected by a moderate to large effect size. Secondly, our findings tentatively suggest that targeting one specific problem such as procrastination can also potentially reduce other mental health complaints such as depression or stress, even if the intervention wasn't aimed at these problems specifically. However, it must be noted that spontaneous remission or regression to the norm cannot be ruled out in our study, given our single-arm design. Further research utilizing a control group is needed to draw conclusions on intervention effectiveness.

Lastly, we examined whether guidance by trained psychology students was acceptable. Our findings indicate sufficient

| | | | | |
|---|---|---|---|---|
| | | | | satisfaction with the guidance, which is comparable to other guided, internet-based, or face-to-face interventions [74,80,81]. |
| Limitations | 19 | Discuss limitations of the study, taking into account sources of potential bias or imprecision. Discuss both direction and magnitude of any potential bias | 12-14 | **Strengths and limitations** A main strength of this study is the comprehensiveness of assessment. When examining acceptability and feasibility of new e-health interventions, studies mainly focus on participant satisfaction and usability. While these were the main outcomes of the present study, we also explored changes to procrastination and several other related mental health complaints. Given that procrastination is linked to mental health issues such as depression, social anxiety, stress, and low self-esteem [13,14,16,17], this study provides valuable insight into how treating procrastination may also impact mood, stress and quality of life. Additionally, we employed post-test as well as follow-up measures. This allowed us to explore both short- and longer-term changes to these mental health complaints. |

However, a major limitation of this study is that we cannot draw any conclusions about the effectiveness of the intervention on student mental health due to lack of a control group. While this feasibility study was not designed to evaluate effectiveness, we did report pre-post change scores on secondary outcomes. While we found positive changes on all the measures, none of these can be directly attributed to the intervention given the lack of a control group. Regression to the mean or spontaneous remission offer alternative explanations for the improvements we found. Moreover, the observed effects may be inflated due to selective study attrition. Although a formal bias analysis falls outside the scope of this paper, we acknowledge that the true change may be smaller than reported. Therefore, in future work we plan to conduct a randomized controlled trial (RCT) that includes a suitable comparison condition (e.g., waitlist control, treatment-as-usual, or active placebo). This will allow us to more rigorously evaluate intervention effectiveness and account for non-specific factors

such as spontaneous remission and placebo effects.

A third limitation of this study regards the sample. During the recruitment process, all students of participating universities were offered access to the intervention. However, not all students are equally likely to be interested in or willing to sign up the intervention, which means our findings are not directly generalizable to the total student population. For example, the majority of participants in the present study was Dutch and female, which limits our understanding of intervention feasibility for other genders and nationalities. Per April 2025, 44,3% percent of university students in the Netherlands were male and 27.1% were international [82,83], compared to 25.2% and 39.2% respectively in our sample. Our sample seems sufficiently diverse in terms of nationality, however, male students are underrepresented. A possible approach for future research is to first try and gain a deeper understanding of why male students in the Netherlands are less

likely to make use of the intervention. Prior research has found that male students may perceive less benefits of and hold more stigma-related attitudes regarding mental health help-seeking [84]. Additional interviews with this target demographic could yield specific, actionable insights on how to increase the uptake of the 'GetStarted' intervention. Secondly, targeted marketing efforts could be made. For example, colleges host many events for specific groups. These events can be a great opportunity to create awareness of the intervention amongst certain groups. Similarly, online marketing efforts such as social media posts could target certain demographics. A recommendation for future recruitment is to aim for between 40% and 45% male participants. A more diverse study sample in further research on the intervention would greatly benefit the generalizability of future findings.

Lastly, the high attrition is a limitation in the present study. Less than half of participants who started the intervention completed the post-

measure, and about a third completed the six-month follow up. Though we found no differences in the baseline characteristics of study completers and non-completers, we cannot rule out any unmeasured confounders. This results in a risk of attrition bias, meaning that 'differences between people who leave a study and those that continue can be the reason for any observed effect and not the intervention itself' [85]. Future research should employ strategies to reduce attrition, such as calling participants as a reminder or offering a financial incentive, in order to avoid attrition bias. Both reminder-based approaches and financial incentives have been shown to improve retention in online intervention trials [86,87].

A strength of this study is our exploration of student e-coaches as the source of guidance. To the best of our knowledge, this study was the first to investigate acceptability of guidance offered by trained psychology students. Prior research has shown that guided internet interventions are more effective

than unguided treatment . However, mental health resources such as properly trained professionals are scarce. It is of paramount importance to find scalable and cost-effective alternatives to licensed psychologist so that people in need can get timely mental health care. Clinical psychology students are required as well as eager to gain hands-on experience during their studies, which makes them a promising potential resource. This study shows that participants of our e-health intervention are satisfied with the guidance offered by student coaches, and tapping into this more readily available resource means people in need can get more or quicker access to mental health support.

Finally, a last strength of this study is the scalability of our approach. First in terms of the guidance offered by e-coaches. In the present study, e-coaches were involved as a part of their curriculum (i.e. a 'mini-internship'), meaning they did receive study credits but not financial compensation. This greatly adds to the scalability of our

approach. While supervising more e-coaches does require slightly more resources in terms of man-hours, this investment is negligible compared to costs involved with utilizing mental health professionals.

Second, the digital platform used to deliver the intervention was specifically developed with scalability in mind. The platform was created by the Caring Universities Project, which is affiliated with the World Health Organization’s World Mental Health International College Student Initiative (WMH-ICS) [51]. We currently offer access to eleven digital interventions including 'GetStarted' and adding additional interventions incurs minimal additional cost. Participating institutions pay a membership fee in exchange for unlimited access to all interventions. Importantly, there is no cap on the number of students who can enroll. Although there are some ongoing maintenance costs associated with platform upkeep, these remain relatively modest, especially in light of the wide range of interventions and the large

| | | | | |
|---|---|---|---|---|
| | | | | number of students that can be reached. Additionally, given the non-commercial nature of Caring Universities, the per-institution fee decreases as additional universities join the initiative, while platform costs remain stable. This model offers substantial potential for large-scale dissemination and implementation. |
| Interpretation | 20 | Give a cautious overall interpretation of results considering objectives, limitations, multiplicity of analyses, results from similar studies, and other relevant evidence | 14 | This study demonstrates promising outcomes for the feasibility and acceptability of "GetStarted"; an e-health intervention targeting procrastination specifically designed for college students guided by psychology students. Participants reported high satisfaction with the intervention and found the system usable. Adherence rates were acceptable given the nature of the intervention (self-help), the topic (procrastination) and the target group (students). While most participants did not finish the intervention, on average they still completed a large part of the intervention. Participants showed a moderate to large decrease in procrastination, a small to moderate decrease in depression and stress, |

| | | | |
|---|---|---|---|
| | | | and a small to moderate increase in quality of life between pre- and posttest. However, these results must be interpreted with caution given the lack of control group in the current study design. Lastly, participants were sufficiently satisfied with the guidance of student e-coaches.<br><br>**Conclusions**<br>The internet-based, student-guided intervention "GetStarted" targeting procrastination appears to be acceptable and feasible for college students in the Netherlands. The intervention could potentially also reduce complaints in the areas of procrastination, mood and stress. Future research should determine possible intervention effectiveness using a control group, as well as explore strategies to address high study attrition. |
| Generalisability | 21 | Discuss the generalisability (external validity) of the study results | A third limitation of this study regards the sample. During the recruitment process, all students of participating universities were offered access to the intervention. However, not all students are equally likely to be interested in or willing to sign up the intervention, |

which means our findings are not directly generalizable to the total student population. For example, the majority of participants in the present study was Dutch and female, which limits our understanding of intervention feasibility for other genders and nationalities. Per April 2025, 44,3% percent of university students in the Netherlands were male and 27.1% were international [82,83], compared to 25.2% and 39.2% respectively in our sample. Our sample seems sufficiently diverse in terms of nationality, however, male students are underrepresented. A possible approach for future research is to first try and gain a deeper understanding of why male students in the Netherlands are less likely to make use of the intervention. Prior research has found that male students may perceive less benefits of and hold more stigma-related attitudes regarding mental health help-seeking [84]. Additional interviews with this target demographic could yield specific, actionable insights on how to increase the uptake of the 'GetStarted' intervention. Secondly,

targeted marketing efforts could be made. For example, colleges host many events for specific groups. These events can be a great opportunity to create awareness of the intervention amongst certain groups. Similarly, online marketing efforts such as social media posts could target certain demographics. A recommendation for future recruitment is to aim for between 40% and 45% male participants. A more diverse study sample in further research on the intervention would greatly benefit the generalizability of future findings.

Lastly, the high attrition is a limitation in the present study. Less than half of participants who started the intervention completed the post-measure, and about a third completed the six-month follow up. Though we found no differences in the baseline characteristics of study completers and non-completers, we cannot rule out any unmeasured confounders. This results in a risk of attrition bias, meaning that 'differences between people who leave a study and those that continue can be the reason for any

| | | | | |
|---|---|---|---|---|
| | | | | observed effect and not the intervention itself' [85]. Future research should employ strategies to reduce attrition, such as calling participants as a reminder or offering a financial incentive, in order to avoid attrition bias. Both reminder-based approaches and financial incentives have been shown to improve retention in online intervention trials [86,87]. |
| **Other information** | | | | |
| Funding | 22 | Give the source of funding and the role of the funders for the present study and, if applicable, for the original study on which the present article is based | 14-15 | The Caring Universities Project is funded by the Vrije Universiteit Amsterdam, Leiden University, Maastricht University, Utrecht University, Erasmus University Rotterdam, University of Amsterdam, Inholland University of Applied Sciences, Rotterdam University of Applied Sciences, and Avans University of Applied Sciences. |

*Give information separately for cases and controls in case-control studies and, if applicable, for exposed and unexposed groups in cohort and cross-sectional studies.

**Note:** An Explanation and Elaboration article discusses each checklist item and gives methodological background and published examples of transparent reporting. The STROBE checklist is best used in conjunction with this article (freely available on the Web sites of PLoS Medicine at http://www.plosmedicine.org/, Annals of Internal Medicine at http://www.annals.org/, and Epidemiology at http://www.epidem.com/). Information on the STROBE Initiative is available at www.strobe-statement.org.
